# Characterization of the gut microbiota in urinary calculi patients with preoperative urinary tract infection

**DOI:** 10.3389/fcimb.2025.1417403

**Published:** 2025-02-28

**Authors:** Hao Chen, Jing Yuan, Hongmin Zhou, Xiangcheng Zhan, Yuchen Gao, Bowen Chen, Nuer Aihemaiti, Xiao Xu, Yunze Dong, Shuai Liu, Yanhua Chen, Ding Liu, Tiancheng Xie, Yunfei Xu

**Affiliations:** Department of Urology, Shanghai Tenth People’s Hospital, School of Medicine, Tongji University, Shanghai, China

**Keywords:** gut microbiota, urinary tract infection, urolithiasis, serum metabolome, 16S rRNA

## Abstract

**Background:**

Urinary tract infection is one of the most common comorbidities of urinary stones. Disorders of gut microbiota can affect various infectious diseases and the formation of the stones. Therefore, alterations in the gut bacteria profile may be a potential risk factor for the development of infections in patients with urinary tract stones.

**Methods:**

We conducted a retrospective study to analyze the association of urinary tract infections with gut microbiota and serum metabolism in patients with stones.

**Results:**

Patients with urolithiasis were predominantly in combination with diabetes mellitus (11.4% vs. 20%) and hypertension (36.4% vs. 50%). There were no statistically significant differences in hematological and urinary parameters. Compared to negative patients, IL-17A was significantly higher in the positive group (25.0 vs 21.1 pg/ml p = 0.038). The majority of pathogens detected in urine cultures were urease-negative bacteria, and urease-positive bacteria accounted for 15% of the total number of patients. We analyzed the community composition of the two groups of patients and found a significant difference in their β-diversity (p = 0.025), suggesting that dysbiosis of the gut bacteria may be associated with the combination of urinary tract infections in urolithiasis. For identification of crucial bacteria, we found changes in the abundance of both *Intestinibacter* (p = 0.036) and *Dialister* (p = 0.039), and abundance of *Intestinibacter* was positively correlated with IFN-α, IL-12P70 (p<0.05), and especially IL-17A (p<0.01), which may result from differences in translational, ribosomal structural and biosynthetic functions in stone patients (p < 0.05).

**Conclusion:**

Urolithiasis with gut dysbiosis developed a higher incidence of urinary tract infections, which may be associated with the increasing of *Intestinibacter* and affect the expression of IL-17A by translational, ribosomal structural and biosynthetic function.

## 1 Introduction

Urolithiasis is a common disease with a prevalence of 5%-20% worldwide, 7%-13% in the US, 5%-9% in the Europe, and 1%-5% in the Asia (Sorokin et al., 2017), with a progressive increased incidence over the last decades (Romero et al., 2010), especially in developed countries (Bauza et al., 2018). Furthermore, the rate of recurrence of urolithiasis in Asia is about 6%-17% after 1 year, 21%-53% after 3-5 years, and 60%-80% for the lifetime (Liu et al., 2018). The direct and indirect costs associated with the treatment of urolithiasis impose a heavy burden on the health care system, with the annual economic cost of urolithiasis estimated to be more than $5 billion in the United States (Saigal et al., 2005).

As one of the most common comorbidities of urinary stones, urinary tract infections (UTI) have affected 150 million people around the world each year (Stamm and Norrby, 2001). Patients with UTI combined with urinary stones are also exposed to an increased risk of complications or cure failure (Geerlings, 2016). They are often administrated empirically or with unregulated antibiotics before sensitivity tests are available, leading to the emergence of drug-resistant bacteria, and further increasing the burden of treatment and decreasing the possibility of recovery (Nicolle, 2006). Urinary stones complicating UTI are caused metabolic stones, associated with *Escherichia coli*, and urinary stones caused by UTI are usually infectious stones, associated with the urease-producing gram-negative organisms (Espinosa-Ortiz et al., 2019; Wang et al., 2024). The bacterial spectrum of complicated UTI in China is characterized by a decreased proportion of *Escherichia coli* and an increased proportion of Extended Spectrum Beta-Lactamases (ESBLs) producing bacterium and *Enterococcus (* Qiao et al., 2013). The risk of treatment-related infections and sepsis is higher in patients with infected stones, especially in combination with immunocompromised or anatomically abnormal urethra or diabetes mellitus (Lieske et al., 2006; Zanetti et al., 2008; Stasinou et al., 2017). Therefore, reducing the risk or improving the symptoms of infections in patients with urinary stones is crucial for the healthcare system currently.

The gut bacteria changes with the internal environment, related to various physiological functions, such as digestion, metabolism and immunity (Adak and Khan, 2019; Gomaa, 2020). The inflammatory conditions resulted from imbalance of gut bacteria has been identified to affect the development of various diseases, such as infectious (Chen et al., 2023), metabolic (Chen et al., 2023), psychological diseases (Sasso et al., 2023) and even tumors (Kostic et al., 2013). As the largest immune organ, the gastrointestinal tract is colonized by gut bacterium that modulate immunity, maintain the intestinal mucosal barrier and defend against pathogens (Gilbert et al., 2018). It has been demonstrated that alterations in the diversity of the gut microbiome contribute to the development of infections through various mechanisms, including the proliferation of toxin-producing bacteria (Hyoju et al., 2019), the release of pro-inflammatory factors (Schirmer et al., 2016), and the reduction of beneficial metabolites (Adelman et al., 2020). Similarly, when infection is confirmed, the gut bacterial flora will be found to imbalance, leading to an increased susceptibility to end-organ dysfunction (Shimizu et al., 2011; Miller et al., 2021). The gut microbiota has been previously reported to be associated with many infectious diseases such as COVID-19 (Zhang et al., 2023), tuberculosis (Yu et al., 2023), respiratory pseudomonas aeruginosa infection (Forestier et al., 2008) and sepsis (Bongers et al., 2023). Additionally, the gut microbiome can influence the formation of urinary stones in multiple mechanisms, including intestinal oxalate uptake and crystalloid formation (Stern et al., 2016; Tang et al., 2018; Miller et al., 2022). However, we found that there is an absence of studies that investigated whether there is a correlation between stone-related complicated urinary tract infections and the diversity of the gut microbiota. Therefore, using 16S rRNA sequencing, we designed a retrospective study to profile the gut microbiome of patients with urinary stones who developed preoperative urinary tract infections. Consequently, we discovered significant differences in the gut microbiome between patients with stone-combined urinary tract infections and patients without infection. These results extended our understanding of stone-combined urinary tract infections and provided a foundation for the prevention, diagnose, and treatment of stone-combined urinary tract infections.

## 2 Materials and methods

### 2.1 Population

The study complied with the Declaration of Helsinki (2008), and was approved by the ethics committee of Shanghai Tenth People’s Hospital (approved number was 21K105), and informed consent was obtained from all patients who participated in the study.

The analysis of gut microbiota included 64 patients with urinary stones admitted to the Shanghai Tenth People’s Hospital affiliated with Tongji University from October 2022 to March 2023 (20 patients were diagnosed with combined urinary tract infections, and 44 patients were not combined with urinary tract infections). Inclusion criteria were as follows (1) age ≥18 years; (2) urinary stones diagnosed by color Doppler ultrasound or CT examination. Exclusion criteria were as follows (1) comorbidity with gastrointestinal diseases; (2) antibiotics and probiotics administered in the last 3 months; (3) comorbidity with non-urinary tract infections; and (4) failure of fecal DNA quality test. Definition of urinary tract infection: positive urine culture and clinical manifestations, such as urinary frequency, urgency, painful urination, or fever and pain in the renal region. In particular, positive urine culture was defined as: urinary fungal culture ≥ 10^3^ cfu/ml or bacterial colony count of clean mid-stream urine >10^5^ cfu/ml for women and >10^4^ cfu/ml for men, or bacterial colony count >10^4^ cfu/ml in all patients’ catheterized retained urine specimens.

### 2.2 Study design

Based on the comorbidities of urinary tract infection, 64 patients with urinary stones were divided into the positive group (20 patients) and the negative group (44 patients). We collected basic information about the patients, cytological indicators and biochemical analyses of hematology and urology, characteristic of urine culture, and levels of inflammatory factors in order to analyze the comparability of the infected and non-infected groups, the characteristics of the gut microbiota profile and the major differential bacteria, and to explore the correlation between the differential microbiota and the indicators related to urinary tract infections.

### 2.3 Sample collection and 16S rRNA analysis

#### 2.3.1 DNA extraction and PCR amplification

Total microbial genomic DNA was extracted from fecal samples using the PF Mag-Bind Stool DNA Kit (Omega Bio-tek, Georgia, U.S.). The extracted DNA was then verified by agarose gel electrophoresis and a NanoDrop^®^ ND-2000 spectrophotometer (Thermo Scientific Inc., USA).

The hypervariable region V3-V4 of the bacterial 16S rRNA gene were amplified with primer pairs 515F (5’-GTGCCAGCMGCCGCGG-3’) and 806R (5’- GGACTACHVGGGTWTCTAAT-3’) (Caporaso et al., 2011) by an ABI GeneAmp^®^ 9700 PCR thermocycler (ABI, CA, USA). All samples were amplified in triplicate. The PCR product was extracted from 2% agarose gel and purified.

#### 2.3.2 Illumina MiSeq sequencing

Purified amplicons were pooled in equimolar amounts and paired-end sequenced on an Illumina PE300 platform (Illumina, San Diego, USA) according to the standard protocols by Majorbio Bio-Pharm Technology Co. Ltd. (Shanghai, China).

#### 2.3.3 Amplicon sequence processing and analysis

After demultiplexing, the resulting sequences were quality filtered with fastp (0.19.6) (Chen et al., 2018) and merged with FLASH (v1.2.7) (Magoč and Salzberg, 2011). Then the high-quality sequences were de-noised using DADA2 (Callahan et al., 2016) plugin in the Qiime2 (Bolyen et al., 2019) pipeline with recommended parameters, which obtains single nucleotide resolution based on error profiles within samples. DADA2 denoised sequences are usually called amplicon sequence variants (ASVs). To minimize the effects of sequencing depth on alpha and beta diversity measure, the number of sequence from each sample was rarefied by minimum number of sample sequences, which still yielded an average Good’s coverage of 99.9%. Taxonomic assignment of ASVs was performed using the Naive bayes consensus taxonomy classifier implemented in Qiime2 and the SILVA 16S rRNA database (v138). The metagenomic function was predicted by PICRUSt2 (Phylogenetic Investigation of Communities by Reconstruction of Unobserved States) (Douglas et al., 2020) based on ASV representative sequences.

Bioinformatic analysis of the gut microbiota was carried out using the Majorbio Cloud platform (https://cloud.majorbio.com). Based on the ASVs information, rarefaction curves and alpha diversity indices including Chao1 richness, Shannon index and Good’s coverage were calculated with Mothur v1.30 (Schloss et al., 2009). The similarity among the microbial communities in different samples was determined by principal coordinate analysis (PCoA) based on Bray-curtis dissimilarity using Vegan v2.4.3 package. The linear discriminant analysis (LDA) effect size (LEfSe) (Segata et al., 2011) (http://huttenhower.sph.harvard.edu/LEfSe) was performed to identify the significantly abundant taxa (phylum to genera) of bacteria among the different groups (LDA score > 2, P < 0.05).

### 2.4 Statistical analysis

Statistical analysis was performed by R 4.3.1. Normally distributed data were described as mean ± standard deviation and non-normally distributed data were described as maximum and minimum. Count data were described as frequency and percentage, compared using the chi-square test, and measured data were compared using the t-test or Wilcoxon signed rank-sum test, with two-sided p<0.05 defined as a statistically significant difference. At the genus level, we plotted joint ROC curves using relative abundance of flora and specific serological indicators. Calculations of odds ratios and their 95% confidence intervals were done to determine the strength of the association between risk factors and outcomes. The data merging method was logistic regression.

## 3 Results

### 3.1 Characteristics of patients

A total of 64 patients with urinary stones were collected, including 20 patients combined with urinary tract infections and 44 patients without infections. As shown in **Table 1**, no significant differences were found between the two groups in terms of demographic characteristics. The average age of the infected and non-infected groups was 59.5 and 63.0 years respectively, and both were predominantly male (75% vs 60%); Both groups were overweight (BMI ≥24 kg/m^2^) with BMIs of 25.4 (± 3.47) kg/m^2^ and 24.7 (± 3.91) kg/m^2^, respectively. Patients with urinary stones had predominantly comorbidities of diabetes mellitus (11.4% vs 20%) and hypertension (36.4% vs 50%). Additionally, nearly half of the patients suffered from left-sided stones (43.2% vs 45.0%). However, no statistically significant differences were found in either hematological or urine indicators. As for inflammatory factors, we found that compared with negative patients, IL-17A was significantly higher in the positive group (25.0 vs 21.1 pg/ml p = 0.038). Nevertheless, no significant differences were found for the other inflammatory factors (**Table 2**).

**Table 1:** Baseline-Characteristics of patients.

Baseline-Characteristics	Negative	Positive	P.value
	(N=44)	(N=20)	
Basic information			
Age (year)	59.5 (32.0, 85.0)	63.0 (33.0, 85.0)	0.40
Sex(Male)	33 (75.0%)	12 (60.0%)	0.22
Position of urinary stone			0.93
Left	19 (43.2%)	9 (45.0%)	
Right	13 (29.5%)	5 (25.0%)	
Both	12 (27.3%)	6 (30.0%)	
Diabetes	5 (11.4%)	4 (20.0%)	0.44
Hypertension	16 (36.4%)	10 (50.0%)	0.30
Body Mass Index (kg/m^2^)	25.4 (± 3.5)	24.7 (± 3.9)	0.51
Hematological parameters			
Hemoglobin (g/L)	138.0 (77.0, 168.0)	137.0 (95.0, 158.0)	0.53
White Blood Cell (*10^9^/L)	7.3 (3.2, 19.4)	7.1 (1.1, 14.7)	0.13
C-reactive protein (mg/L)	3.8 (0.5, 142.0)	2.5 (0.5, 36.9)	0.26
Platelet (*10^9^/L)	239.0(92.0, 524.0)	250.0 (100.0, 325.0)	0.53
Neutrophils%	68.4 (± 11.5)	63.7 (± 10.8)	0.12
Lymphocyte%	23.4 (2.7, 52.5)	27.4 (11.9, 59.2)	0.09
Creatinine (μmol/L)	78.1 (34.3, 437.0)	74.1 (47.9, 181.0)	0.63
Urea (μmol/L)	6.4 (3.9, 18.6)	5.50 (3.9, 13.1)	0.87
Uric acid (μmol/L)	388.0 (± 99.3)	362.0 (± 82.9)	0.29
Urinalysis			
WBC counts/HP*	13.0 (0, 505.0)	14.0 (0, 959.0)	0.82
RBC counts/HP*	30.0 (0, 34300.0)	7.00 (0, 16200.0)	0.51
PH	6.0 (5.0, 7.5)	6.3 (5.0, 7.5)	0.29
Leukocyte esterase	n=40	n=18	0.37
Negative	25 (62.5%)	9 (50.0%)	
Positive	15 (37.5%)	9 (50.0%)	
Nitrite	n=41	n=18	0.46
Negative	39 (95.1%)	16 (88.9%)	
Positive	1 (2.4%)	2 (11.1%)	
Strongly positive	1 (2.4%)	0 (0%)	

Values are number (percentage), median (Min, Max) or Mean ± SD.

*WBC,White blood cell; RBC, Red blood cell.

**Table 2:** The difference of inflammatory factors.

Inflammatory factor (pg/ml)	Positive	Negative	P.value
	(N=20)	(N=44)	
IL2	1.38 (0.310, 6.00)	1.49 (0.150, 4.76)	0.818
IL4	1.86 (0.0100, 8.12)	1.65 (0.0100, 10.0)	0.678
IL5	0.891 (± 0.573)	1.02 (± 0.584)	0.431
IL6	30.4 (2.54, 2500)	8.73 (1.86, 1220)	0.152
IL8	36.5 (5.81, 2500)	59.7 (2.56, 2500)	0.524
IL1b	2.14 (0.0100, 97.6)	2.28 (0.0100, 42.7)	0.77
IL17A	25.0 (2.16, 340)	21.1 (0.260, 50.0)	0.038*
IL10	3.23 (0.820, 289)	2.50 (0.0400, 19.9)	0.282
IFNa	2.38 (0.0100, 11.8)	1.76 (0.0100, 11.8)	0.289
TNFa	4.92 (0.230, 172)	7.91 (0.0100, 154)	0.318
IL12P70	2.67 (0.520, 11.2)	2.66 (0.0100, 10.3)	0.549
IFNr	3.37 (1.44, 14.8)	2.38 (0.220, 6.04)	0.105

Values are median (Min, Max) or Mean ± SD.

Asterisks (*) represent significant differences at P < 0.05.

**Figure 1:**
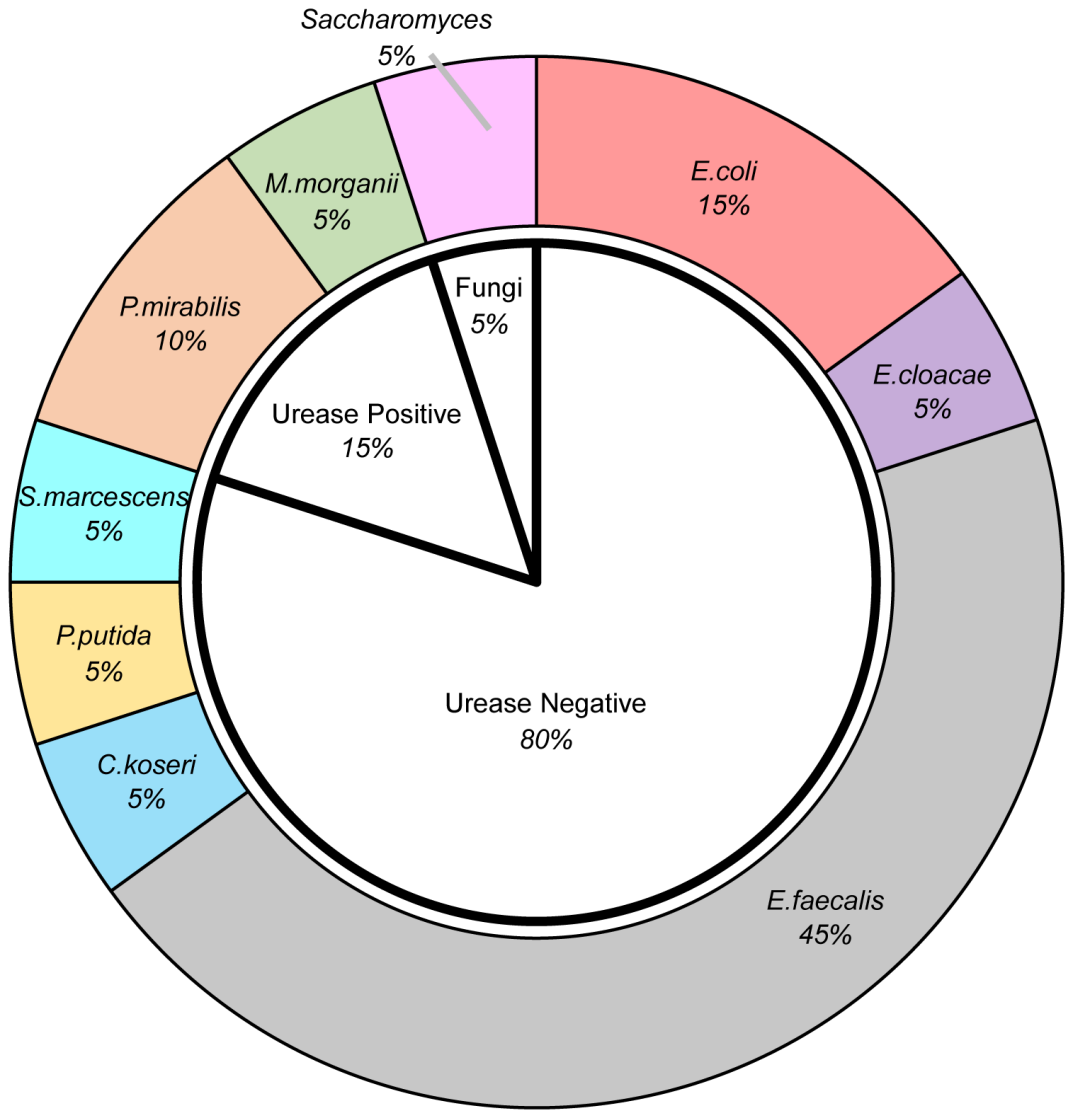
The pie diagram demonstrated the distribution of pathogens detected in urine culture. *E.coli, *Escherichia coli*; E.cloacae, *Enterobacter Cloacae*; E.faecalis, *Enterococcus faecalis*; C.koseri, *Citrobacter Koseri*; P.putida, *Pseudomonas putida*; S.marcescens, *Serratia marcescens*; P.mirabilis, *Proteus mirabilis*; M.morganii, *Morganella morganii*.

Among the patients with comorbidity of urinary tract infection, when it comes to the pathogens, as shown in **Figure 1**, the majority of pathogens detected in urine culture were urease-negative bacteria, predominantly *Enterococcus faecalis*, accounting for 45% of the patients. Other urease-negative pathogens included *Escherichia coli*, *Enterobacter Cloacae, Citrobacter Koseri*, *Pseudomonas putida*, and *Serratia marcescens*. Urease-positive bacteria accounted for 15% of the patients, including *Morganella morganii*, *Proteus mirabilis.* There was also a case infected with *Saccharomyces*.

### 3.2 Holistic differences in gut microbiota profiles

**Figure 2:**
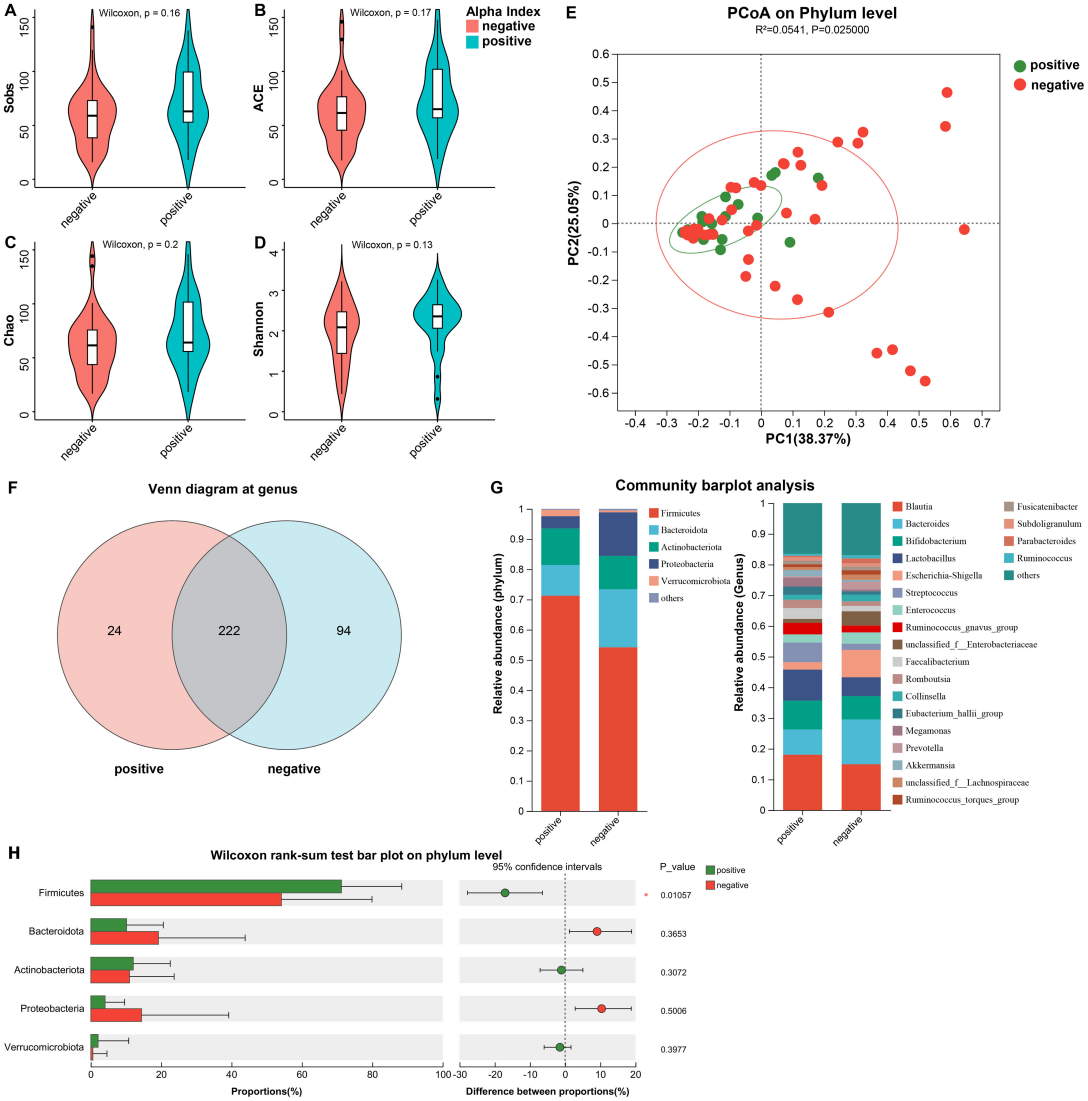
The differences in community diversity and the structure of the microbiota between the two groups. **(A–D)** The α diversity (including Sobs, ACE, Chao, and Shannon) were not found significant alterations. **(E)** The PCoA on Phylum level demonstrated a significant difference in β-diversity. **(F)** Venn diagram depicted the differences at genus. **(G)** The distribution of major phylum and genus in both groups. **(H)** Wilcoxon rank-sum test bar plot on phylum level. *ACE, Abundance-based coverage estimator; PCoA, principal coordinate analysis.

Through sequencing of the fecal 16S rRNA from the patients, we initially investigated the differences in community diversity between the two groups and noticed that the positive group developed significant changes in the structure of the microbiota. In contrast to the negative group, the α diversity (including Sobs, ACE(Abundance-based Coverage Estimator), Chao, and Shannon) were not found significant alterations in the positive group (**Figure 2**), which suggested that the community diversity and abundance of gut bacteria were similar between the two groups. However, using principal coordinate analysis (PCoA), we found a significant difference in β-diversity which could demonstrate the composition of microflora (**Figure 2E**, p = 0.025), which suggested that the altered profile of the gut bacteria perhaps correlated with the comorbidity of urinary tract infection in urinary stone patients.

For further exploration of the contribution to the differences in β-diversity, we demonstrated the profiles of community composition of the two groups at the level of phylum and genus. We found that at the genus, the characteristics of gut microbiota distribution between the two groups was significantly different. Twenty-four genera were unique to the positive group, accounting for 9.76%, and almost one-third were unique to the negative group (29.75%), with up to 94 genus (**Figure 2F**). **Figure 2G** illustrated the percentage of major phylum and genus in both groups. **Figure 2H** shows the significant difference between the two groups at the phylum level. For patients with comorbid infections, the proportion of *Firmicutes* increased (71.24% vs. 54.17% p = 0.011), while the *Bacteroidota* decreased (10.17% vs. 19.23% p = 0.365). Therefore, we subjected *Firmicutes*/*Bacteroidota* to Wilcoxon rank-sum test and obtained F/B (11.43 (3.61~149.13) vs 8.33 (1.47~51.84), p=0.241). Thus we concluded that there was no significant difference in F/B between the two groups. At the genus, the infected group was dominated by *Brucella* (18.06%), *Lactobacillus* (10.12%) and *Aspergillus* (4.08%), while the negative group was dominated by *Brucella* (14.95%), *Lactobacillus* (14.58%) and *Aspergillus* (14.43%), and the differences in the above genus were not statistically significant. The other gut communities included *Bifidobacterium* (9.41% vs 7.64%), *Streptococcus* (6.41% vs 2.01%) and *Escherichia-Shigella* (2.39% vs 8.93%).

### 3.3 Identification of critical bacteria and their correlation with infection

**Figure 3:**
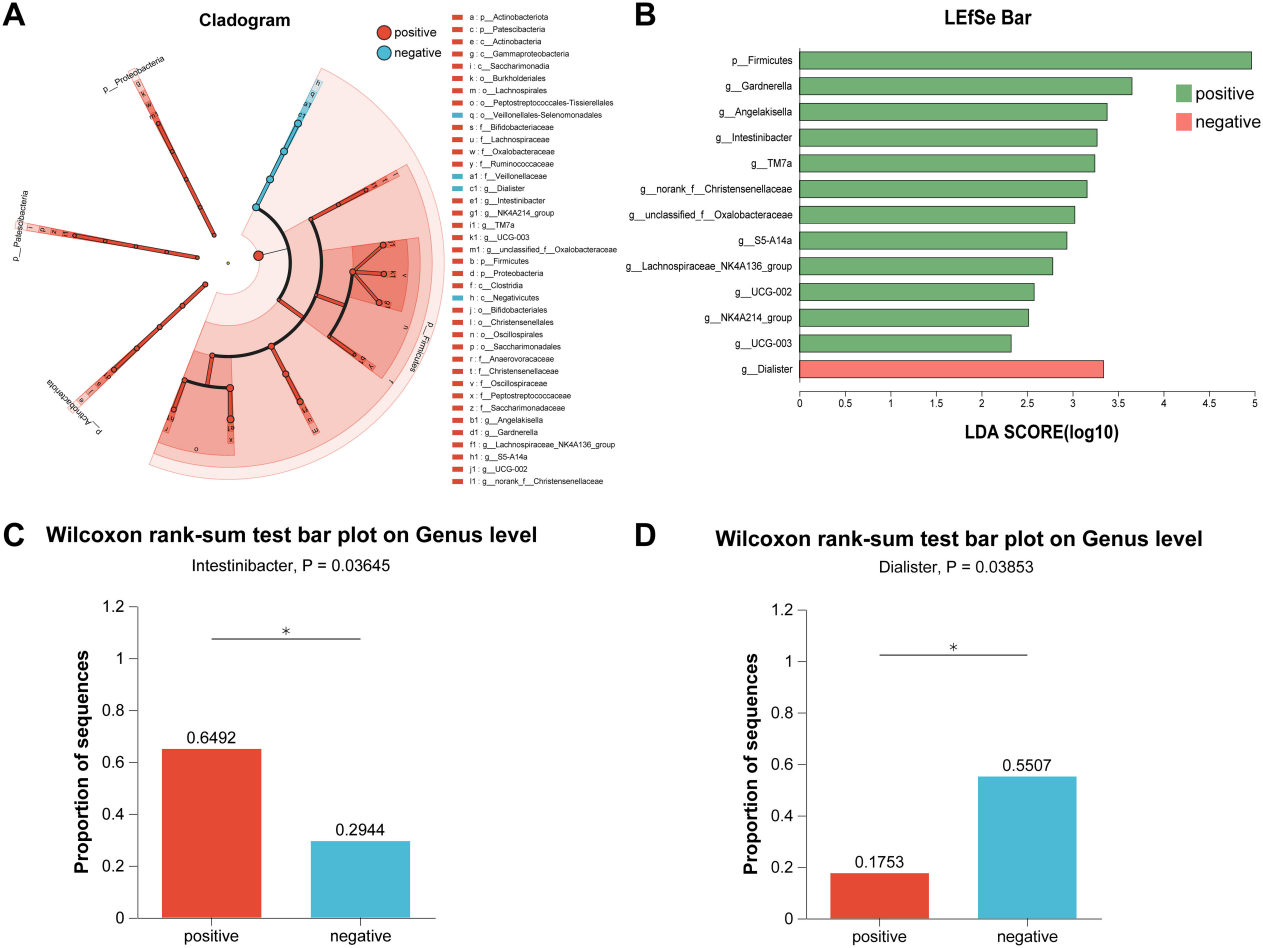
Identification of crucial bacteria by LEfSe analysis. **(A)** Cladogram of LEfSe analysis. **(B)** Bar of LEfSe analysis. **(C)** The bar plot of Wilcoxon rank-sum test on Genus level for *Intestinibacter*. **(D)** The bar plot of Wilcoxon rank-sum test on Genus level for *Dialister*. *LEfSe, Linear discriminant analysis Effect Size; LDA, Linear discriminant analysis.

Holistically, dysregulation of the gut microbiota composition profoundly influenced urinary tract infections in patients with urinary stones; therefore, to further identified the cardinal bacteria in the differential gut microbiota profiles, we performed Linear discriminant analysis Effect Size (LEfSe) from the phylum to the genus with LDA (Linear discriminant analysis) score > 2 (**Figures 3A, B**). **Figure 3A** shows the LEfSe multilevel species level tree. As depicted in **Figure 3B**, compared to non-infected individuals, the abundance of *Firmicutes, Gardnerella, Angelakisella, Intestinibacter, Christensenellaceae*, and *Oxalobacteraceae* were increased in infected patients, suggesting that these communities may critically contribute to the pathogenesis of urinary tract infections in patients with stones. *Dialister* was observed to be increased in negative patients, indicating that the bacteria may act as an antagonist in the process of urinary tract infections. Remarkably, both *Intestinibacter* and *Dialister* were ranked as top 50 in terms of abundance, and the analysis of differences between groups also revealed that abundance has been changed for both *Intestinibacter* (p = 0.036), and *Dialister* (p = 0.039) (**Figures 3C, D**).

**Figure 4:**
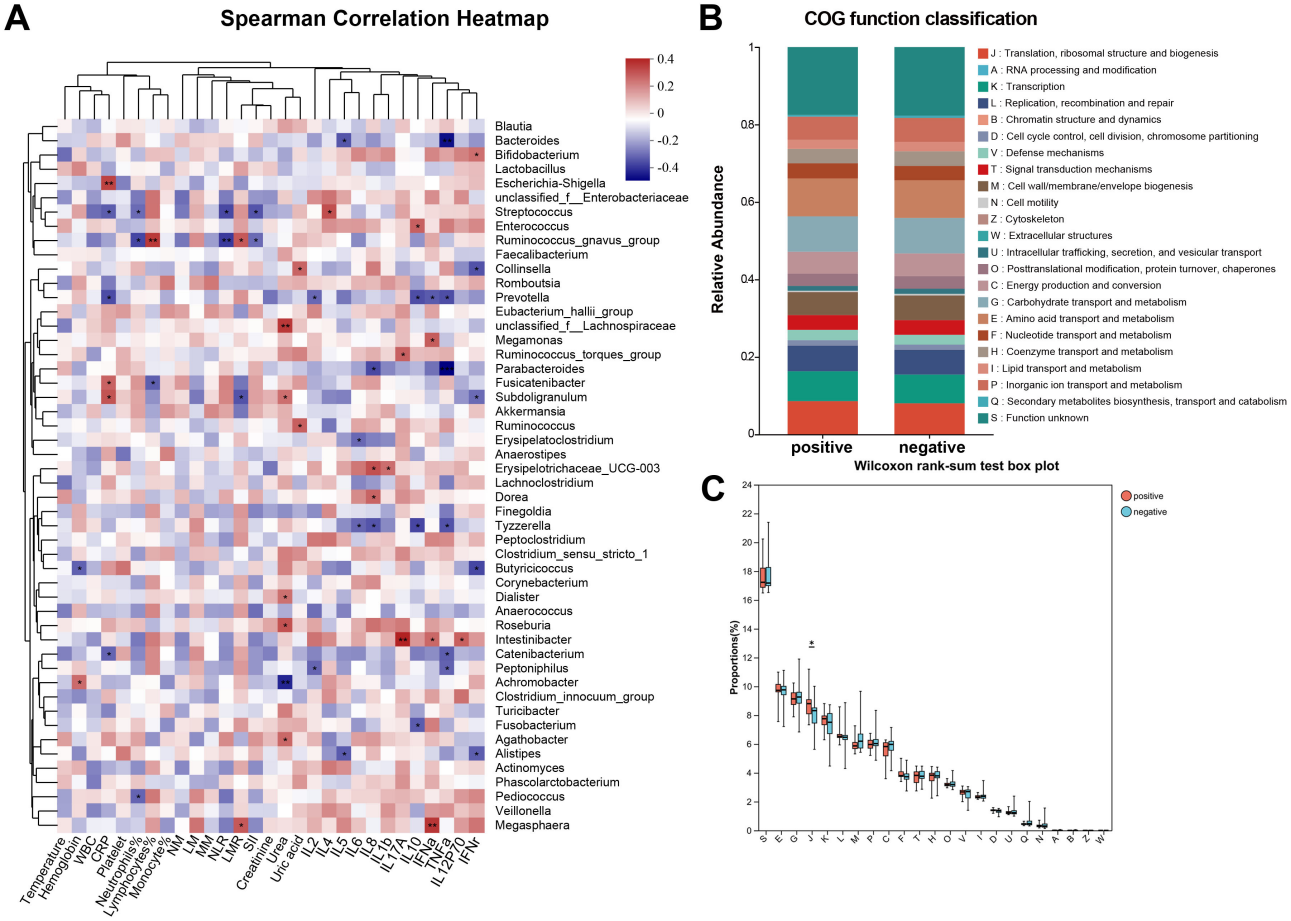
Correlation analysis and function verification between critical bacteria and the pathogenic process of urinary tract infections. **(A)** Spearman correlation analysis between critical bacteria and indicators of infection. **(B)** Classification statistics of COG functions. **(C)** Box plot of Wilcoxon rank-sum test for the function ofCOG functions. *COG, Clusters of Orthologous Groups; *J (Translation, ribosomal structure and biogenesis).

**Figure 5:**
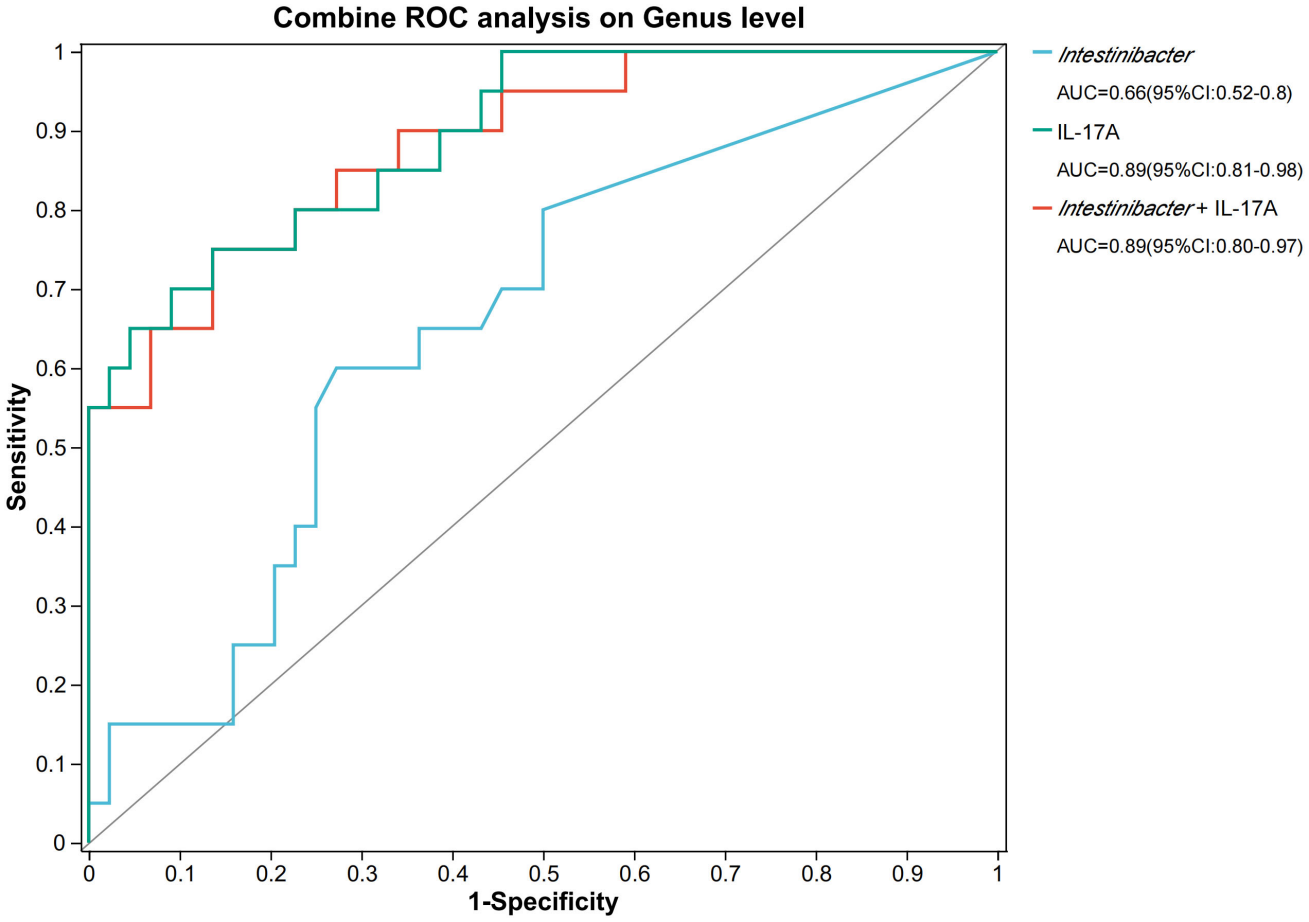
The ROC analysis on IL-17A and *Intestinibacter*. *AUC, area under the curve; ROC, Receiver operating characteristic.

Therefore, we investigated the effects of gut bacteria on urinary tract infections in stone patients by Spearman correlation analysis and further verified the essential roles of *Intestinibacter* and *Dialister* in the pathogenic process of urinary tract infections (**Figure 4A**). Surprisingly, for the top 50 genera of abundance, *Intestinibacter’s* abundance was positively correlated with IFN-α, IL-12P70 (p<0.05), and especially IL-17A (p<0.01), in accordance with the significant elevation of IL-17A in the infected group (p=0.038). However, except for increased serum urea (p<0.05), *Dialister* was not found to have an effect on the other infection-related markers. In addition, P*revotella, Tyzzerella and Catenibacterium* were also negatively associated with various inflammatory factors (p<0.05), suggesting a potential protective role against urinary tract infections. A total of 23 pathways were predicted based on PICRUSt2 (**Figure 4B**). As shown in **Figure 4C**, abundance statistics of COG (Clusters of Orthologous Groups) functions suggested that the influence of the gut microbiota on the development of urinary tract infections may result from differences in translational, ribosomal structural and biosynthetic functions in stone patients (p < 0.05). The remaining pathways were not statistically different. At the Genus level, we set up a combined ROC analysis of *Intestinibacter* relative abundance and IL-17A, the data merging method was logistic regression (**Figure 5**). As shown in **Figure 5**, the area under the curve (AUC) for the fitting of urinary tract infection in stone patients with the IL-17A was 0.89, the AUC for the abundance of *Intestinibacter* was 0.66 and the AUC when combining the abundance of *Intestinibacter* and IL-17A was 0.89. This indicated that the IL-17A can well predict the occurrence of urinary tract infection in urolithiasis, and the infection prediction using the abundance of *Intestinibacter*, is supported but less efficient. Perhaps there are still other factors besides intestinal that influence the expression and secretion of IL-17A. Collectively, we concluded that the increased *Intestinibacter* abundance was closely associated with the pathogenesis of urinary tract infections in patients with stones, and it has been shown that IL-17A was significantly elevated in patients with urinary tract infections. Therefore, considering the correlation analysis of *Intestinibacter* was highly concordant with the baseline characteristics in terms of IL-17A, *Intestinibacter* may induce the pathogenesis of urinary tract infections in patients with stones by affecting the expression or secretion of IL-17A.

## 4 Discussion

The prevalence of urolithiasis has gradually increased over the past decades and more than half of the patients will suffer from recurrence (Bichler et al., 2002). Additionally, urinary tract infections, a common comorbidities of urinary stones, also cause a huge medical burden to the health care system (Ziemba and Matlaga, 2017). The emergence of both drug-resistant bacteria and treatment-related sepsis have presented overwhelming challenges in the management of stone-complicated urinary tract infections (Wang et al., 2024), therefore, it will be a major task to explore the pathogenesis of stone-complicated urinary tract infections so as to reduce the risk of infections for urinary stone patients and control the symptoms of infections. Numerous previous studies have described the relationship between gut microbiota and immunity, infectious diseases, and stone formation, whereas we depicted the different gut microbiota landscapes between infected and non-infected patients with urinary stones by 16S rRNA sequencing, and found a significant difference in the microbiota structure between the infected and non-infected groups, with apparent dysbiosis in patients with the infected group. Furthermore, we found that *Intestinibacter* might influence the development of urinary tract infections critically in patients with stones by affecting the expression and secretion of IL-17A.

It has been demonstrated that alterations in the gut bacterial flora increase susceptibility to sepsis (Forestier et al., 2008; Adelman et al., 2020; Bongers et al., 2023), suggesting that the changes in the enrichment and composition of the gut community, may affect the development of urinary tract infections in urinary stone patients. Previous literature has reported that the *Firmicutes*/*Bacteroidota* (F/B) ratio is associated with the maintenance of homeostasis, and that variations in this ratio can lead to various pathologies, such as obesity and intestinal inflammation (Shen et al., 2018; Abenavoli et al., 2019; Stojanov et al., 2020). In our study, there was a significant difference in *Firmicutes* between the two groups, but there was no difference in *Bacteroidota*, nor was there a difference in F/B. We conclude that concomitant urinary tract infections in patients with urolithiasis are not related to the value of F/B. In accordance with the results we obtained, the alterations in the bacterial composition, such as the enrichment of *Intestinibacter*, may lead to dysregulation of immunophysiology, which in turn may have an impact on the development of infections. IL-17A is considered to increase the chemotactic activity of neutrophils and is associated with infectious and autoimmune diseases. In autoimmune diseases, IL-17A is mainly produced by pathological Th 17 cells, whereas in urinary tract infections it is synthesized by Tγδ lymphocytes, which can recognize bacterial ligands independently of MHC restriction (Sivick et al., 2010; Chamoun et al., 2020). The relationship between IL-17A and urinary tract infections has been proved in animal models. Mice deficient in IL-17A exhibit impaired bacterial clearance and reduced macrophage and neutrophil influx in the bladder and kidneys (Asadi Karam et al., 2022). Previous studies have also mentioned that serum levels of IL-17A were shown to be increased in patients with urinary tract infections compared with non-infection patients (Wojciuk et al., 2022). The gut bacterial flora is associated with the activation of many immunocytokines, including IL-17A (Geva-Zatorsky et al., 2017). Moreover, IL-17A has been confirmed to be relevant to infections, autoinflammatory diseases, and renal fibrosis (Kolls and Lindén, 2004; Peroumal and Biswas, 2023). In the results of our study, based on the baseline characteristics of the patients, we found a significant difference in IL-17A between the infected and control groups in patients with stones.

In our study, by combining metabolism, gut bacteria composition and urinary tract infections, we demonstrated that the enrichment of *Intestinibacter* may affect IL-17A and thus result to a greater susceptibility to urinary tract infections in patients with stones. However, some of the findings in our study should also be discussed. Notably, there was no significant difference in inflammation-related indicators between the two groups of patients, such as CRP and white blood cell counts, which was probably attributed to the association between stones and inflammatory reaction (Sakhaee, 2022; Capolongo et al., 2023). Therefore, we should recruit more participants and divide them into four groups: patients with urine culture-positive stones, patients with stones not combined with urinary tract infections, patients without stones but with urinary tract infections, and normal people, and compare the differences in inflammatory indicators and intestinal bacteria characteristics between the groups. Furthermore, we can compare whether there are also differences in the gut microflora between patients infected with different bacteria, which may require multicenter studies with larger sample sizes. The clinical problem we focused on trying to address was to reduce the complication rate of urinary tract infections in patients with urolithiasis. The ROC curve model was fitted to the incidence of urinary tract infections occurring in patients with urolithiasis. We limited our interest to patients with urinary tract stones. With the inclusion of healthy individuals, it is also important to consider whether intestinal flora affects stone formation, and mutual confounders between urolithiasis and urinary tract infections are not conducive to analysis. The current study controls for variables and investigate the relationship between urinary tract infections and intestinal flora, probably it is rather the results will be more convincing. Besides, the 16S rRNA sequencing we used could not cover all species, the composition and functional analysis of microorganisms still need more refinements. The pathophysiological mechanisms and signaling pathways of altered gut microbiota on inflammation and infection in patients with stone co-infections still need to be explored, and therefore future studies may require the application of macro-genomic sequencing (MGS) and other metabolomics analyses.

Urolithiasis holds a high rate of incidence and recurrence, and urinary tract stone co-infections are also challenging to cure due to the emergence of drug-resistant bacteria and their complexity, and the risk of progression of sepsis and systemic inflammatory response syndrome (SIRS). Hence, we showed that stone patients with disordered gut microbiota tended to be exposed to urinary tract infections, which may be associated with the *Intestinibacter* and IL-17A. Therefore, it is recommended to take various measures to regulate the gut microflora of stone patients, which may reduce the risk of infection, alleviate the symptoms of infection, and increase the possibility of recovery.

In conclusion, we conclude that the intestinal flora was altered in the urolithiasis combined with urinary tract infection group, as characterized by significant differences in composition, with increased proportions of *Firmicutes*. There was an increase in *Intestinibacter* and a decrease in *Dialister* among the top 50 abundances in the positive group. The significant roles of *Intestinibacter* and *Dialister* in the pathogenesis of urinary tract infection were further verified by Spearman analysis. IL-17A was obviously elevated in the positive group and directly correlated with the abundance of *Intestinibacter*. COG functions showed that the influence of intestinal microbiota on the development of urinary tract infection may be related to Translation, ribosomal structure and biogenesis. The area under the curve (AUC) for the fitting of urinary tract infection in stone patients with the IL-17A was 0.89, the AUC for the abundance of *Intestinibacter* was 0.66 and the AUC when combining the abundance of *Intestinibacter* and IL-17A was 0.89. This may be due to the causal correlation between *Intestinibacter* and IL-17A. Therefore, we considered that *Intestinibacter* may induce the pathogenesis of urinary tract infection in stone patients by affecting the expression or secretion of IL-17A. Future studies may be able to further elucidate the causality and related pathways of urolithiasis combined with urinary tract infection, intestinal flora, and IL-17A. This may require more sample size, MGS, and more animal experiments.

## Data Availability

The data presented in the study are deposited in the National Center for Biotechnology Information (NCBI) repository, accession number PRJNA1102608.
